# Synthesis and Characterization of Calcium Hydroxyapatite from Waste Phosphogypsum

**DOI:** 10.3390/ma18122869

**Published:** 2025-06-17

**Authors:** Elzbieta Jursene, Laura Michailova, Simona Jureviciute, Zivile Stankeviciute, Inga Grigoraviciute, Aivaras Kareiva

**Affiliations:** Institute of Chemistry, Vilnius University, Naugarduko 24, LT-03225 Vilnius, Lithuania; elzbieta.jursene@chgf.stud.vu.lt (E.J.); laura.michailova@chgf.stud.vu.lt (L.M.); simona.jureviciute@chgf.stud.vu.lt (S.J.); zivile.stankeviciute@chf.vu.lt (Z.S.); inga.grigoraviciute@gmail.com (I.G.)

**Keywords:** calcium hydroxyapatite, dissolution–precipitation synthesis, phosphogypsum waste, bioceramic materials

## Abstract

In this study, phosphogypsum waste collected from a factory dump in Kedainiai, Lithuania, was used for the first time as a starting material in the dissolution–precipitation synthesis of high-quality bioceramic calcium hydroxyapatite (Ca_10_(PO_4_)_6_(OH)_2_; CHA). The CHA powders were synthesized using the dissolution–precipitation method, employing phosphogypsum in four different conditions: untreated, dried at 100 °C, dried at 150 °C, and annealed at 1000 °C. Various phosphorus sources were used in the CHA synthesis process: Na_2_HPO_4_; a mixture of Na_2_HPO_4_ and NaH_2_PO_4_; or a combination of Na_2_HPO_4_, NaH_2_PO_4_, and NaHCO_3_. These mixtures were allowed to react at 80 °C for 48 h, 96 h, 144 h, and 192 h. X-ray diffraction (XRD) analysis revealed slight variations in the synthesized products depending on the specific starting materials used. Fourier transform infrared spectroscopy (FTIR) was conducted to confirm the structural characteristics of the synthesized CHA samples. The surface microstructure of the synthesized CHA samples differed notably from that of the raw phosphogypsum. All synthesized CHA samples exhibited Type IV nitrogen adsorption–desorption isotherms with H3-type hysteresis loops, indicating the presence of mesoporous structures, typically associated with slit-like pores or aggregates of plate-like particles. To the best of our knowledge, an almost monophasic CHA has been fabricated from phosphogypsum waste for the first time using a newly developed dissolution–precipitation synthesis method. A key challenge in the high-end market is the development of alternative synthesis technologies that are not only more environmentally friendly but also highly efficient. These findings demonstrate that phosphogypsum is a viable and sustainable raw material for CHA synthesis, with promising applications in the medical field, including the production of artificial bone implants.

## 1. Introduction

Synthetic calcium hydroxyapatite (Ca_10_(PO_4_)_6_(OH)_2_; CHA) is widely used as a bioceramic material to treat bone defects due to its chemical similarity to bone minerals, as well as its favorable biocompatibility and plasticity [1,2,3,4,5,6,7,8]. However, the quality of synthetic biomaterials largely depends on the overall characteristics and properties of the synthesized powders, including their density, purity, phase composition, crystallinity, particle size and distribution, morphology, and specific surface area [9,10]. Therefore, all of the aforementioned properties of bioceramics are highly influenced by the processing conditions, which play a crucial role in determining the crystallinity, crystal shape, crystal size, size distribution, and phase purity of the resulting powders.

Several methods have been employed for the synthesis of CHA. Calcium hydroxyapatite powders have been synthesized by using precipitation [11,12,13,14], hydrothermal [15,16,17,18,19], conventional solid-state reaction [14,19,20,21,22], mechanochemical [14,19,23,24,25,26], and sol–gel [4,9,27,28,29] methods. Overall, precipitation, hydrothermal, and sol–gel techniques can produce high-purity CHA powders with small particle sizes. However, important process variables including solution concentration, Ca/P ratio, pH, acid or complexing agent addition rate, stirring speed, temperature, reaction time, and atmospheric conditions should be controlled very carefully to obtain a monophasic end product. In standard procedures of solid-state reactions and mechanochemical methods, precursors are first milled and then calcined at very high temperatures, above 1000 °C, resulting in well-crystallized structures. However, it is important to note that powders usually exhibit heterogeneity, and are composed of irregularly shaped micron-sized grains. These methods suffer from the small diffusion of ions during the reactions following the formation of side phases.

Therefore, the synthesis method plays a critical role in controlling the physicochemical properties of CHA, and subsequently influence its mechanical and biological performance. A large number of review papers have been published on the synthesis of CHA from various natural sources, highlighting their advantages and disadvantages, their applications, and the essential properties of CHA for therapeutic applications [30,31,32,33,34]. Recent methods for extracting CHA from natural sources—such as mammalian, aquatic, or marine organisms, shells, eggshells, plants and algae, wood, animal bones, and minerals—have also been described. The use of plant-derived CHA at surgical sites can provide antifungal and antibacterial effects, helping to prevent infections in the affected area.

It was demonstrated that mollusk-derived hydroxyapatite is a widely available, cost-effective, sustainable, and low-impact biomaterial [35,36,37]. The results of previous studies [38,39] suggest that pufferfish teeth could be a natural alternative source for dental applications. The natural CHA produced from cow bone through ultrasound treatment followed by a calcination process at various temperatures and derived from marine resources could also be used for biomedical purposes [40,41].

An enormous number of scientific articles have been published on the potential of obtaining CHA from various wastes [42,43,44]. The utilization of eggshell waste as a raw material for the synthesis of CHA was suggested in several publications. In the initial preparation stage, calcium oxide was derived from eggshell waste and employed as a calcium precursor in order to prepare CHA via chemical precipitation with phosphoric acid [45,46]. CHA from eggshell wastes was also synthesized through mechanochemical activation and solid-sate reaction processes [47,48]. It was also demonstrated that shrimp shell waste, mussel shell waste, and other seafood shells could be a source of calcium to produce CHA [49,50,51,52]. Moreover, the potential use of bovine bone waste, fish waste, food products, sulphite waste, marble wastes, and other materials for the fabrication of CHA was suggested elsewhere [53,54,55,56,57]. Recently, we demonstrated for the first time that phosphogypsum waste could be successfully used as a precursor for the fabrication of CHA [58]. The company *Lifosa*, based in Lithuania, specializes in manufacturing phosphate-based fertilizers such as diammonium phosphate, monoammonium phosphate, and monocalcium phosphate. It produces phosphoric acid in house by processing apatite, which is then used in subsequent fertilizer production stages. One of the by-products generated during phosphoric acid synthesis is phosphogypsum. The factory site has accumulated a substantial amount of phosphogypsum waste, estimated at approximately 45 million tons. Although phosphogypsum is often classified as non-hazardous and is considered harmless to human health and the environment, its chemical composition can vary depending on the source of the phosphate raw material—apatite—used in the production process. To produce CHA and address potential disadvantages, such as those associated with solid-state reactions, appropriate synthesis methods must be proposed. A major challenge, particularly at the high end of the market, is to develop alternative synthesis technologies that are not only more environmentally friendly but also efficient and effective. The aim of this study is to optimize the dissolution–precipitation synthetic approach for the synthesis of high-quality CHA from phosphogypsum waste. In this paper, we present the results of the systematic characterization of an environmentally friendly dissolution–precipitation approach to CHA synthesis. The findings demonstrate that phosphogypsum is a viable and sustainable raw material for CHA synthesis, with promising applications for the production of artificial bone implants.

## 2. Materials and Methods

Waste phosphogypsum obtained from the company *Lifosa*, disodium hydrogen phosphate (Na_2_HPO_4_, 98%, Merck, Darmstadt, Germany), sodium dihydrogen phosphate (NaH_2_PO_4_, 99%, Merck, Darmstadt, Germany), and sodium hydrogen carbonate (NaHCO_3_, 989%, Merck, Darmstadt, Germany) were used as starting materials for the fabrication of calcium hydroxyapatite (Ca_10_(PO_4_)_6_(OH)_2_; CHA) powders via a dissolution–precipitation reaction.

In order to assess the influence of the starting material on the formation of CHA, some phosphogypsum samples were prepared, which were taken from different parts of the raw material. CHA powders were subsequently synthesized by using the dissolution–precipitation method with untreated phosphogypsum, while other samples were dried at 100 °C or 150 °C, or annealed at 1000 °C. In the CHA synthesis process, a 1.00 g portion of waste phosphogypsum (i.e., untreated and subjected to drying at 100 °C or 150 °C, or annealing at 1000 °C) was placed in the reaction vessel. It was then mixed with various reagent solutions serving as phosphorus sources: (A) 100.0 mL of 1.00 M Na_2_HPO_4_ solution; (B) a mixture containing 50.0 mL of each 1.00 M Na_2_HPO_4_ and 1.00 M NaH_2_PO_4_; or (C) a mixture containing 50.0 mL of each 1.00 M Na_2_HPO_4_, 1.00 M NaH_2_PO_4_, and 1.00 M NaHCO_3_. The mixtures were left to react at 80 °C for 48, 96, 144, and 192 h. After synthesis, the liquid phase was decanted, and the resulting powders were rinsed with 500 mL of hot (~80 °C) deionized water, followed by several additional rinses using 250 mL of room-temperature deionized water. Finally, the vacuum-filtered product was dried at 80 °C for 2 h.

Phosphorus starting materials were selected based on findings reported in the literature. For instance, a mixture of Na_2_HPO_4_ and NaH_2_PO_4_ has been successfully employed in the synthesis of magnesium whitlockite via the dissolution–precipitation method [59]. Similarly, octacalcium phosphate blocks have been fabricated through a dissolution–precipitation reaction using Na_2_HPO_4_ as the phosphorus source [60]. Bone apatite is a form of carbonated calcium hydroxyapatite that typically contains 6–9 mass% carbonate within its apatitic structure [61]. Based on this, a mixture of Na_2_HPO_4_, NaH_2_PO_4_, and NaHCO_3_ was also used for the synthesis of calcium hydroxyapatite to better replicate the composition of natural bone apatite.

The prepared samples were characterized by powder X-ray diffraction (XRD) using a Rigaku MiniFlex II diffractometer (Rigaku, Tokyo, Japan) with Cu Kα radiation (λ = 1.541838 Å). The diffraction data were obtained by scanning in the 2θ range of 10–60° at a scan speed of 2°/min. Fourier transform infrared spectroscopy (FT-IR) was performed using an Alpha spectrometer (Bruker, Inc., Ettlingen, Germany) in the wavenumber range from 4000 to 450 cm^−1^, with a resolution of 4 cm^−1^. Product morphology was analyzed using field-emission scanning electron microscopy (SEM, SU-70, Hitachi, Tokyo, Japan). Particle size was evaluated using open-source Fiji (ImageJ2) software by randomly selecting a few hundred particles. Energy-dispersive X-ray (EDX) analysis of the samples was performed using a SEM Hitachi TM 3000 (Tokyo, Japan). The specific surface area was measured by using the Brunauer–Emmet–Teller (BET) method under vacuum for samples degassed at 120 °C with a N_2_ adsorption–desorption isotherm (at 77 K) using a Tristar II instrument (Norcross, GA, USA). The pore size distribution of the materials produced was obtained using the Barrett–Joyner–Halenda (BJH) method.

## 3. Results and Discussion

**Figure 1:**
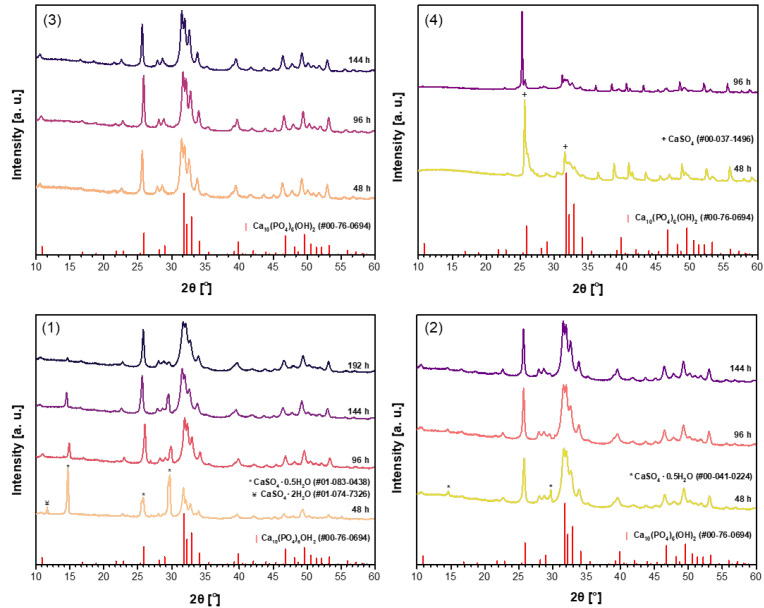
XRD patterns of the CHA samples synthesized from unheated phosphogypsum (**1**), phosphogypsum dried at 100 °C (**2**) or 150 °C (**3**), or phosphogypsum heated at 1000 °C (**4**) using Na_2_HPO_4_ and different durations in the dissolution–precipitation procedure.

The powder XRD patterns of CHA samples synthesized from unheated phosphogypsum, as well as those dried at 100 °C or 150 °C and heated at 1000 °C using Na_2_HPO_4_ (A) in the dissolution–precipitation procedure, are presented in Figure 1.

Evidently, the phase purity of the synthesized samples is dependent on the specific precursor utilized in the synthesis process. The more pronounced CHA phase is observed in the sample synthesized using unheated phosphogypsum (Figure 1(1)) only after a duration of 96 h. However, the diffraction lines of the CaSO_4_·0.5H_2_O secondary phase are visible even after treatment for 144 h. The impurity phase decreases significantly when increasing the reaction time to 192 h. When phosphogypsum dried at 100 °C (Figure 1(2)) was used as the starting material, very similar results are observed. Contrary, when phosphogypsum dried at 150 °C (Figure 1(3)) was used as the calcium source, the monophasic CHA was obtained after treatment for 96 h. With increasing synthesis time, no changes in XRD pattern are observed, indicating that all diffraction peaks belong to the desirable CHA phase. Obviously, the CaSO_4_ phase cannot be used as precursor for the synthesis of CHA by using dissolution–precipitation method (see Figure 1(4)).

**Figure 2:**
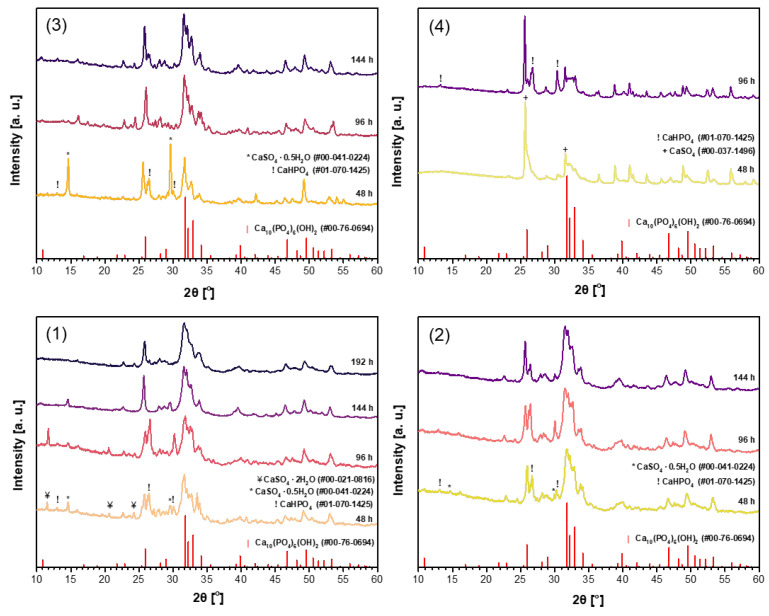
XRD patterns of the CHA samples synthesized from unheated phosphogypsum (**1**), phosphogypsum dried at 100 °C (**2**) or 150 °C (**3**), or phosphogypsum heated at 1000 °C (**4**) using a mixture of Na_2_HPO_4_ + NaH_2_PO_4_ and different durations in the dissolution–precipitation procedure.

**Figure 3:**
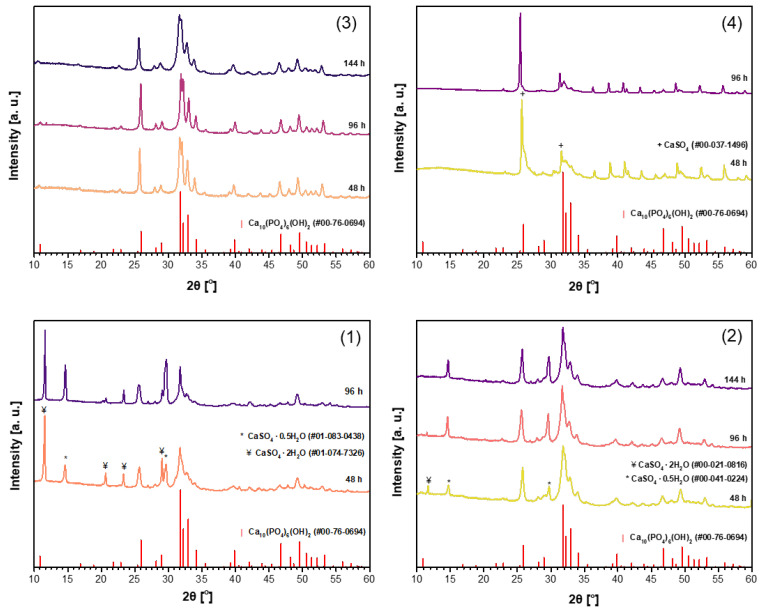
XRD patterns of the CHA samples synthesized from unheated phosphogypsum (**1**), phosphogypsum dried at 100 °C (**2**) or 150 °C (**3**), or phosphogypsum heated at 1000 °C (**4**) using a mixture of Na_2_HPO_4_ + NaH_2_PO_4_ + NaHCO_3_ and different durations in the dissolution–precipitation procedure.

It was demonstrated recently that for the synthesis of magnesium whitlockite (Ca_18_Mg_2_(HPO_4_)_2_(PO_4_)_12_) using the dissolution–precipitation method, a mixture of sodium phosphates (Na_2_HPO_4_ + NaH_2_PO_4_) as the phosphorus source was successfully used [59]. In order to reduce the synthesis time while obtaining a purer product, the same phosphate mixture was used in the synthesis of CHA in this work. Powder XRD patterns of the CHA samples synthesized from the same precursors using a mixture of Na_2_HPO_4_ and NaH_2_PO_4_ (B) in the dissolution–precipitation procedure are shown in Figure 2. Unfortunately, as can be seen from the XRD patterns depicted in Figure 2, the efficiency of CHA production could not be improved. Therefore, a third synthesis route was tested, where phosphogypsum was exposed to three reagents during synthesis, namely Na_2_HPO_4_, NaH_2_PO_4_, and NaHCO_3_ (C). XRD patterns of the resultant products are displayed in Figure 3. XRD analysis results show that the synthesis products are slightly different depending on which starting materials were used in the synthesis protocol. Interestingly, the duration of synthesis decreased to 48 h when CHA was synthesized using the phosphogypsum precursor dried at 150 °C (Figure 3(3)).

In conclusion, the almost polycrystalline single-phase Ca_10_(PO_4_)_6_(OH)_2_ was obtained from waste phosphogypsum by using the dissolution–precipitation synthesis method with the phosphogypsum precursor dried at 100 °C and 150 °C as the Ca source and Na_2_HPO_4_ or a mixture of Na_2_HPO_4_ + NaH_2_PO_4_ + NaHCO_3_ as the P source. These results highlight the influence of both the precursor drying temperature and the choice of phosphorus source on the phase purity of the final apatite product. The purest CHA samples were selected for further characterization. Miller indices have been assigned to the diffraction peaks observed in the XRD pattern of the CHA sample synthesized for 96 h from dried phosphogypsum at 150 °C, using a mixture of Na_2_HPO_4_ + NaH_2_PO_4_ + NaHCO_3_ in the dissolution–precipitation process (Figure 3(3)). Nearly all diffraction lines have been indexed, confirming the formation of monophasic CHA (see Figure S1).

The tentative crystallite sizes for two representative samples were determined by using the Scherrer equation: *τ* = 0.9*λ/Bcosθ*, where τ is the mean crystallite size, λ is the X-ray wavelength, B is the line broadening at half maximum intensity (FWHM) (in radians), and θ is the Bragg angle. The calculated crystallite sizes for the samples obtained from dried phosphogypsum at 150 °C using either Na_2_HPO_4_ alone or a mixture of Na_2_HPO_4_ + NaH_2_PO_4_ + NaHCO_3_ were 59.25 nm and 50.08 nm, respectively.

**Figure 4:**
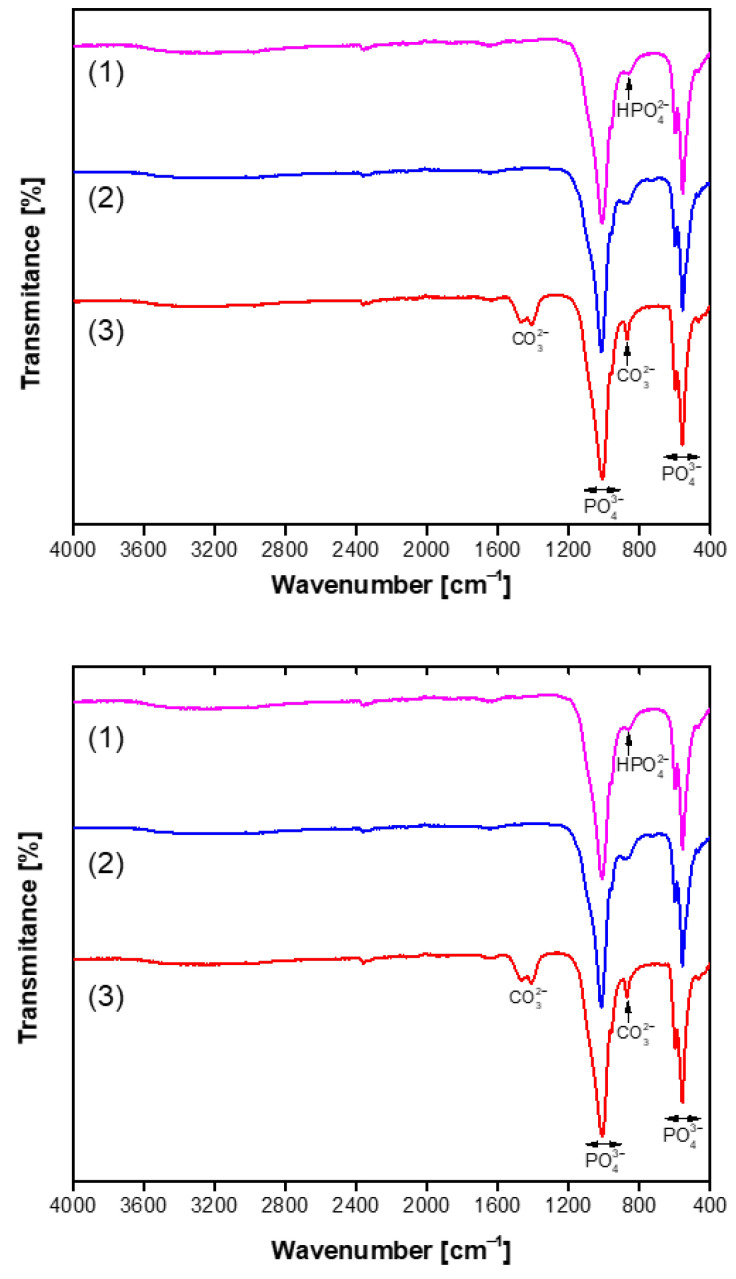
FTIR spectra of CHA samples synthesized from phosphogypsum dried at 100 °C (**bottom**) and dried at 150 °C (**top**) and reacted with different phosphorus sources: Na_2_HPO_4_ (1), Na_2_HPO_4_ + NaH_2_PO_4_ (2), and a mixture of Na_2_HPO_4_ + NaH_2_PO_4_ + NaHCO_3_ (3). The synthesis procedure was carried out for 144 h.

The FTIR spectra in the range of 4000–400 cm^−1^ of the representative and purest CHA samples synthesized for 144 h are presented in Figure 4. The strong absorption bands, which are further subdivided into multiple components within the range of 1170–955 cm^−1^, can be attributed to the distinct vibrational modes associated with the stretching vibrations of the P–O bonds in the PO_4_^3−^ ion [59]. The bands observed at approximately 600–465 cm^−1^ are associated with the bending vibrations of the P–O and O–P–O bonds in phosphate [59,62]. The presence of a broad absorption feature around 869 cm⁻^1^, linked to the P–O(H) vibrational mode of hydrogen phosphate, indicates a deficiency of calcium in the structure of the produced apatite-type material. The stretching vibrations of the O–H group in the CHA structure were detected as a broad absorption band in the 3575–3440 cm^−1^ region, along with a weaker band observed around 1680–1650 cm^−1^. In the case when a mixture of Na_2_HPO_4_ + NaH_2_PO_4_ + NaHCO_3_ was used in the dissolution–precipitation synthesis procedure, the FTIR spectra of the resulting CHA samples revealed distinct absorption bands in the 1550–1360 cm^−1^ region at 1468 cm⁻^1^, 1456 cm⁻^1^, and 1417 cm⁻^1^. In biological and synthetic apatites, carbonate can substitute at two primary sites: the phosphate site (known as B-type substitution) or the hydroxyl (OH⁻) site (A-type substitution). The peaks observed in this study are consistent with B-type substitution, which is the most common form in biological apatite and typically results in bands around 1410–1470 cm⁻^1^ [62]. These findings allow us to conclude that in this synthesis route, carbonated hydroxyapatite (Ca_10−x/2_(PO_4_)_6−x_(CO_3_)_x_(OH)_2_) has formed [61,63]. Thus, FTIR analysis was performed to confirm the structural characteristics of the synthesized CHA sample. Moreover, the FTIR results clearly support the conclusions made from the XRD data.

Considering the results obtained, the formation mechanisms of CHA using different phosphorus sources (A), (B), and (C) can be roughly expressed by the following chemical reactions, respectively:

10 CaSO_4_·0.5H_2_O + 6 Na_2_HPO_4_ → Ca_10_(PO_4_)_6_(OH)_2_ +8 NaHSO_4_ + 2 Na_2_SO_4_ + 3 H_2_O (1)

10 CaSO_4_·0.5H_2_O + 4 Na_2_HPO_4_ + 2 NaH_2_PO_4_ → Ca_10_(PO_4_)_6_(OH)_2_ +10 NaHSO_4_ + 3 H_2_O (2)

14 CaSO_4_·0.5H_2_O + 8 Na_2_HPO_4_ + 2 NaH_2_PO_4_ + 6 NaHCO_3_ → Ca_10_(PO_4_)_6_(OH)_2_ + Ca_4_(PO_4_)_4_(CO_3_)_2_(OH)_2_ + 4 NaHSO_4_ + 10 Na_2_SO_4_ + 4 CO + 11 H_2_O (3)

**Figure 5:**
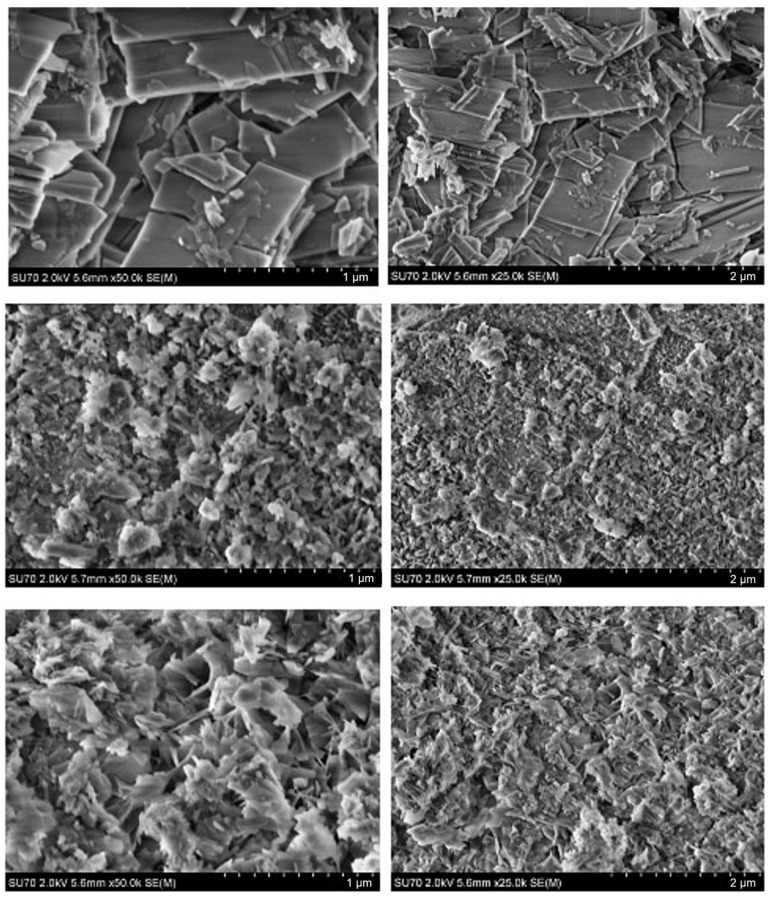
SEM micrographs obtained at different magnifications of the CHA samples synthesized from phosphogypsum dried at 100 °C and reacted with different phosphorus sources: Na_2_HPO_4_ (**bottom**), Na_2_HPO_4_ + NaH_2_PO_4_ (**middle**), and Na_2_HPO_4_ + NaH_2_PO_4_ + NaHCO_3_ (**top**). The synthesis procedure was carried out for 144 h.

SEM micrographs of the CHA samples synthesized from phosphogypsum dried at 100 °C using different phosphorus sources are presented in Figure 5.

The SEM analysis reveals that the morphology of the surfaces of synthesized CHA is dependent on the phosphorus source. The SEM micrographs of the CHA sample synthesized using Na_2_HPO_4_ demonstrate a surface morphology composed of plate-like almost nanosized (~100–300 nm) differently oriented particles with a distinctive shape. The SEM images demonstrate that the surfaces of samples synthesized using the mixture of Na_2_HPO_4_ and NaH_2_PO_4_ are monolithic and predominantly composed of irregularly shaped (flower petals, plates, or rods) particles 50–200 nm in size. All these samples have visible pores on the surface, likely due to the release of gaseous products during synthesis. After NaHCO_3_ was added to the mixture of Na_2_HPO_4_ and NaH_2_PO_4_, a surface microstructure with dominant larger (about 0.5 µm) rectangular plate-like particles was observed. Interestingly, the surface microstructure of obtained the CHA samples is obviously different from that of the initial phosphogypsum, which is composed of larger and differently oriented 20–30 µm in size plate-like crystals and microrods [58].

**Figure 6:**
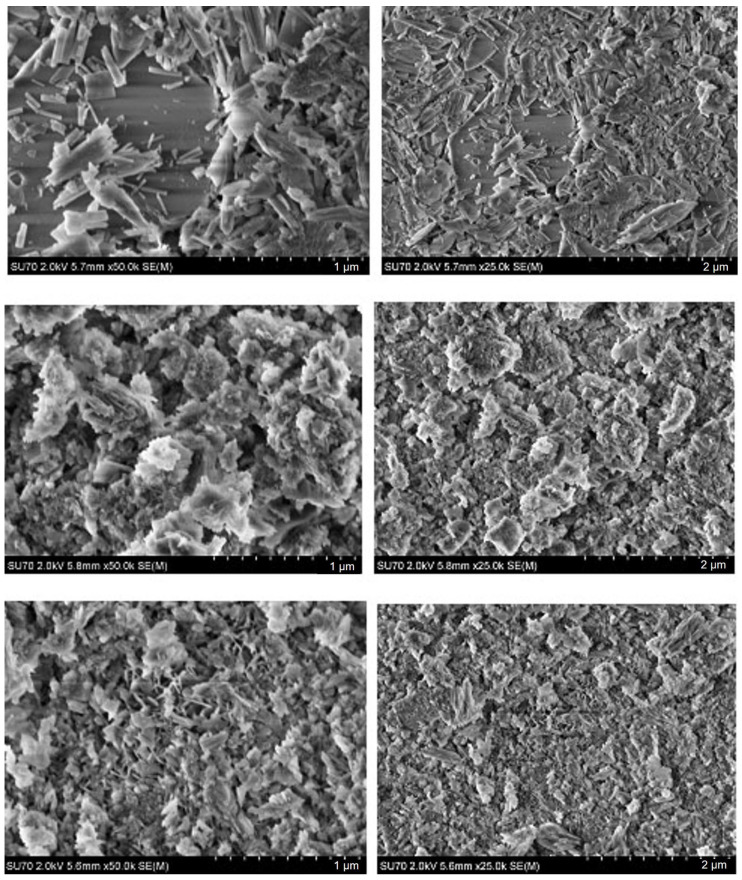
SEM micrographs obtained at different magnifications of the CHA samples synthesized from phosphogypsum dried at 150 °C and reacted with different phosphorus sources: Na_2_HPO_4_ (**bottom**), Na_2_HPO_4_ + NaH_2_PO_4_ (**middle**), and Na_2_HPO_4_ + NaH_2_PO_4_ + NaHCO_3_ (**top**). The synthesis procedure was carried out for 144 h.

SEM micrographs of the CHA samples synthesized from phosphogypsum dried at 150 °C using different phosphorus sources are presented in Figure 6.

It is evident from the SEM images that the microstructural features of the synthesized CHA samples are almost identical to those presented in Figure 5. Thus, surface morphology depends mostly on the phosphorus source (Na_2_HPO_4_, or Na_2_HPO_4_ + NaH_2_PO_4_, or Na_2_HPO_4_ + NaH_2_PO_4_ + NaHCO_3_) used, but not on the initial drying temperature (100 °C or 150 °C) of the phosphogypsum which was used as the starting material. It could be concluded that dissolution–precipitation synthesis processing is responsible for the surface morphology of the final product.

**Figure 7:**
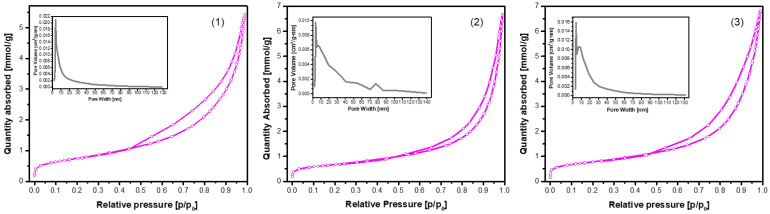
N_2_ adsorption–desorption isotherms of the CHA samples synthesized via the dissolution–precipitation method from phosphogypsum dried at 100 °C and reacted with Na_2_HPO_4_ (**1**), phosphogypsum dried at 150 °C and reacted with Na_2_HPO_4_ (**2**), and phosphogypsum dried at 150 °C and reacted with a mixed solution of Na_2_HPO_4_ + NaH_2_PO_4_ + NaHCO_3_ (**3**). The synthesis procedure was carried out for 144 h. The inset shows the pore size distribution.

The N_2_ adsorption–desorption isotherms of the CHA samples synthesized via the dissolution–precipitation method from phosphogypsum dried at 100 °C and reacted with Na_2_HPO_4_, phosphogypsum dried at 150 °C and reacted with Na_2_HPO_4_, and phosphogypsum dried at 150 °C and reacted with a mixed solution of Na_2_HPO_4_ + NaH_2_PO_4_ + NaHCO_3_ for 144 h are presented in Figure 7.

All samples exhibited Type IV isotherms with H3-type hysteresis loops, a characteristic feature of mesoporous materials with slit-like pores or plate-like particle aggregates. However, a notable variation in surface area, pore volume, and pore distribution was observed, indicative of the impact of synthesis conditions (see Figure 7 and Table 1).

The sample obtained from phosphogypsum dried at 100 °C and subsequently reacted with Na_2_HPO_4_ exhibited a well-developed mesoporous structure, as evidenced by the presence of the highest S_BET_ and S_ext_, in conjunction with a narrow pore size distribution. The close values of S_BET_ and S_ext_ indicate that the majority of the surface area is derived from mesopores. The low Vμ value indicates negligible microporosity. As demonstrated in sample (2), there was a notable similarity in **S_BET_** (58.7 m^2^/g) between the two samples. However, a significant disparity was observed in terms of V_p_ (0.21 cm^3^/g) and micropore contribution (V_μ_ = 0.0040 cm^3^/g). As demonstrated in sample (3), a notably lower S_BET_ (15.9 m^2^/g) and V_p_ (0.067 cm^3^/g) were observed, despite the highest nitrogen uptake being exhibited at high relative pressures. This apparent contradiction can be attributed to the presence of larger mesopores or macropores, which contribute to gas volume but not significantly to surface area. The low external surface area (8.30 m^2^/g) and relatively small V_μ_ (0.0036 cm^3^/g) suggest limited textural development, possibly due to synthesis-related densification or partial pore blockage.

**Figure 8:**
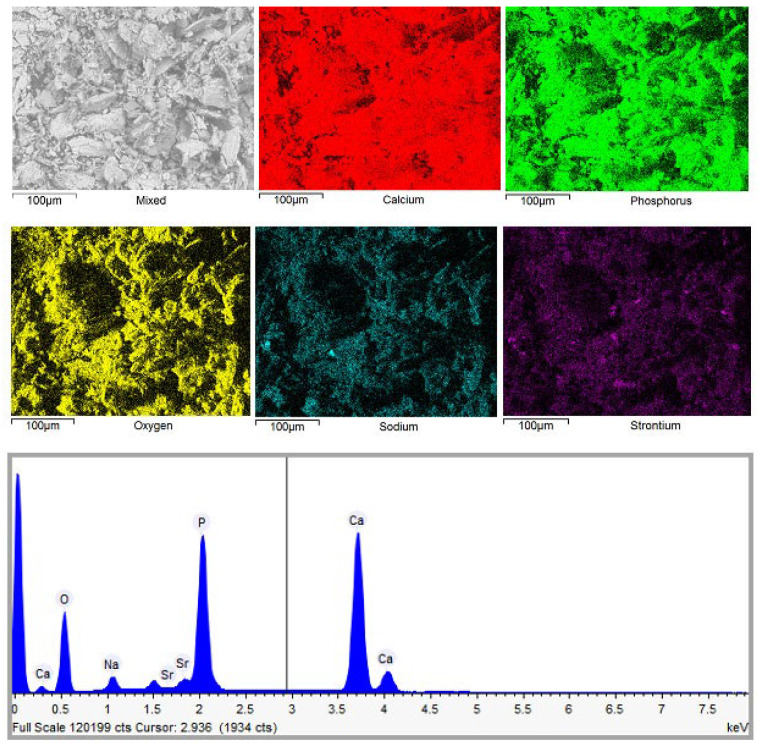
The EDX elemental mapping and spectrum of the CHA sample synthesized via the dissolution–precipitation method from phosphogypsum dried at 150 °C and reacted with a mixed solution of Na_2_HPO_4_ + NaH_2_PO_4_ + NaHCO_3_. The synthesis procedure was carried out for 144 h.

Figure 8 shows elemental mapping of the representative sample composed of plate-like shaped particles.

In all cases, EDX elemental mapping demonstrates that all elements appear to be homogeneously distributed throughout the samples despite the differences in their preparation. The molar ratio of Ca and P in most of the cases corresponds to a ratio 1.67, which is characteristic of the ratio of these elements in calcium hydroxyapatite. A negligible amount of sodium (remaining from starting reagents) and strontium (initial presence in phosphogypsum waste) was also determined in the end products by EDX analysis. Since we did not observe Ca-rich, P-rich, or other metal-rich regions, we can conclude that the almost monophasic CHA could be fabricated by using the dissolution–precipitation synthesis method from phosphogypsum waste.

Finally, high-quality Ca_10_(PO_4_)_6_(OH)_2_ was successfully synthesized from phosphogypsum waste. This approach not only provides a valuable route for producing biomedical CHA but also aligns with the principles of sustainability by converting industrial by-products into high-value materials [64]. Utilizing phosphogypsum, a common waste product from phosphate fertilizer production, helps reduce environmental burden, promotes resource circularity, and supports cleaner production practices [65].

## 4. Conclusions

In this study, the dissolution–precipitation synthetic approach for the synthesis of high-quality calcium hydroxyapatite (Ca_10_(PO_4_)_6_(OH)_2_; CHA) from phosphogypsum waste was optimized. CHA powders were subsequently synthesized via the dissolution–precipitation method using untreated, dried at 100 °C, dried at 150 °C, or annealed at 1000 °C phosphogypsum. In the CHA synthesis process, various phosphorus sources were used (Na_2_HPO_4_; or mixture of Na_2_HPO_4_ and 1.00 M NaH_2_PO_4_; or mixture of 1.00 M Na_2_HPO_4_, 1.00 M NaH_2_PO_4_, and 1.00 M NaHCO_3_). The mixtures were left for 48 h, 96 h, 144 h, and 192 h, allowing the reaction to progress at 80 °C. The phase purity of the synthesized samples was dependent on the specific precursor utilized in the synthesis process. The polycrystalline almost single-phase Ca_10_(PO_4_)_6_(OH)_2_ was obtained from waste phosphogypsum by using the dissolution–precipitation synthesis method when the phosphogypsum precursor dried at 100 °C and 150 °C was used as the Ca source and Na_2_HPO_4_ or a mixture of Na_2_HPO_4_ + NaH_2_PO_4_ + NaHCO_3_ was used as the P source. The SEM analysis reveals that the morphology of the surfaces of synthesized CHA is dependent on the phosphorus source. The SEM micrographs of the synthesized CHA sample using Na_2_HPO_4_ demonstrate a surface morphology composed of plate-like almost nanosized (~100–300 nm) differently oriented particles with a distinctive shape. The SEM images demonstrate that the surfaces of samples synthesized using the mixture of Na_2_HPO_4_ and NaH_2_PO_4_ are monolithic and predominantly composed of irregularly shaped (flower petals, plates, or rods) particles 50–200 nm in size. After NaHCO_3_ was added to the mixture of Na_2_HPO_4_ and NaH_2_PO_4_, the surface microstructure with dominant larger (about 0.5 µm) rectangular plate-like particles was observed. EDX elemental mapping demonstrates that all elements appear to be homogeneously distributed throughout the samples despite the differences in their preparation. The molar ratio of Ca and P in most of the cases corresponds to a ratio 1.67, which is characteristic of the ratio of these elements in calcium hydroxyapatite. In conclusion, the almost monophasic CHA could be fabricated by using the dissolution–precipitation synthesis method with phosphogypsum waste.

## Figures and Tables

**Table 1:** Data derived from N_2_ adsorption–desorption measurements: S_BET_ (specific surface area determined by the BET method), S_ext_ (external surface area), V_μ_ (micropore volume), and V_p_ (total pore volume).

Sample	1	2	3
**>S_BET_ (m^2^/g)**	60.5	58.7	15.9
**S_ext_ (m^2^/g)**	56.4	44.3	8.30
**V_μ_ (cm^3^/g)**	0.0013	0.0040	0.0036
**V_p_ (cm^3^/g)**	0.17	0.21	0.067

## Data Availability

The original contributions presented in this study are included in the article material. Further inquiries can be directed to the corresponding author.

## Supplementary Materials

The following supporting information can be downloaded at: https://www.mdpi.com/article/10.3390/ma18122869/s1, Figure S1: XRD pattern of the CHA sample synthesized for 96 h from dried phosphogypsum at 150 °C using a mixture of Na_2_HPO_4_ + NaH_2_PO_4_ + NaHCO_3_ in the dissolution–precipitation procedure.
