# Supplementary material for: Synthesis and Characterization of Calcium Hydroxyapatite from Waste Phosphogypsum
Source: Materials (Basel). 2025 Jun 17;18(12):2869. doi: 10.3390/ma18122869 (PMC12194978; doi:10.3390/ma18122869)
Supplement: Supplementary file 1 [file materials-18-02869-s001.zip › materials-3671681-supplementary.pdf]

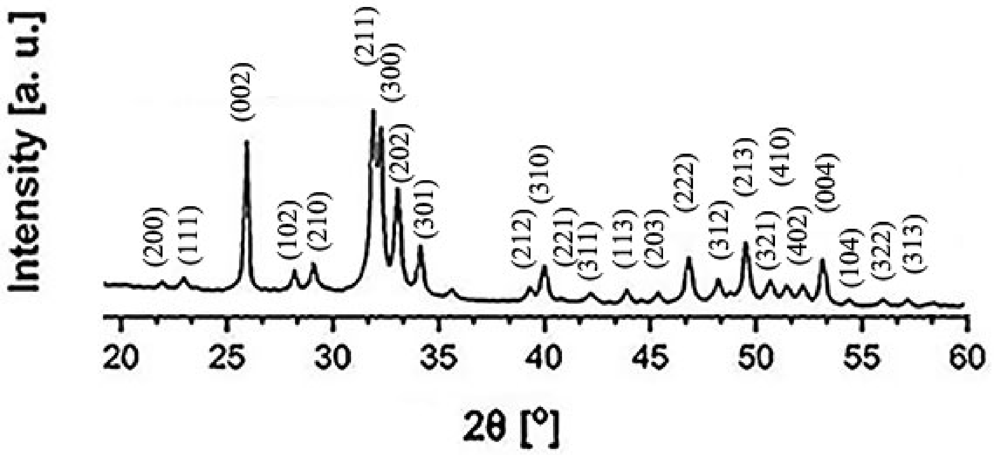

Figure S1. XRD pattern of the CHA sample synthesized for 96 h from dried phosphogypsum at 150 °C using a mixture of Na_2HPO_4 + NaH_2PO_4 + $NaHCO_3$ in the dissolution–precipitation procedure.
